# Fear of COVID-19 for Individuals and Family Members: Indications from the National Cross-Sectional Study of the EPICOVID19 Web-Based Survey

**DOI:** 10.3390/ijerph18063248

**Published:** 2021-03-21

**Authors:** Liliana Cori, Olivia Curzio, Fulvio Adorni, Federica Prinelli, Marianna Noale, Caterina Trevisan, Loredana Fortunato, Andrea Giacomelli, Fabrizio Bianchi

**Affiliations:** 1 Department of Environmental Epidemiology and Disease Registries, Institute of Clinical Physiology, National Research Council, 56124 Pisa, Italy; liliana.cori@ifc.cnr.it (L.C.); fabriepi@ifc.cnr.it (F.B.); 2 Unit of Epidemiology, Institute of Biomedical Technologies, National Research Council, Segrate, 20157 Milan, Italy; fulvio.adorni@itb.cnr.it (F.A.); federica.prinelli@itb.cnr.it (F.P.); 3 Institute of Neurosciences, National Research Council, 35127 Padova, Italy; marianna.noale@in.cnr.it (M.N.); caterina.trevisan.5@studenti.unipd.it (C.T.); 4 Epidemiology and Health Research Laboratory, Institute of Clinical Physiology, National Research Council, 56124 Pisa, Italy; loredana.fortunato@ifc.cnr.it; 5 Infectious Diseases Unit, Department of Biomedical and Clinical Sciences L. Sacco, University of Milan, ASST Fatebenefratelli Sacco, 20157 Milan, Italy; andrea.giacomelli@unimi.it; 6 Institute for Research and Innovation in Biomedicine, National Research Council, 90148 Palermo, Italy

**Keywords:** Coronavirus disease (COVID-19), voluntary respondents, web-based survey, self-reported symptoms, fear, health status, risk perception

## Abstract

The study analyzed the association of the fear of contagion for oneself and for family members (FMs) during the first wave of the COVID-19 pandemic, with demographic and socioeconomic status (SES) and health factors. The study was performed within the EPICOVID19 web-based Italian survey, involving adults from April–June 2020. Out of 207,341 respondents, 95.9% completed the questionnaire (60% women with an average age of 47.3 vs. 48.9 years among men). The association between fear and demographic and SES characteristics, contacts with COVID-19 cases, nasopharyngeal swab, self-perceived health, flu vaccination, chronic diseases and specific symptoms was analyzed by logistic regression model; odds ratios adjusted for sex, age, education and occupation were calculated (aORs). Fear for FMs prevailed over fear for oneself and was higher among women than men. Fear for oneself decreased with higher levels of education and in those who perceived good health. Among those vaccinated for the flu, 40.8% responded they had feelings of fear for themselves vs. 34.2% of the not vaccinated. Fear increased when diseases were declared and it was higher when associated with symptoms such as chest pain, olfactory/taste disorders, heart palpitations (aORs > 1.5), lung or kidney diseases, hypertension, depression and/or anxiety. Trends in fear by region showed the highest percentage of positive responses in the southern regions. The knowledge gained from these results should be used to produce tailored messages and shared public health decisions.

## 1. Background

On 11 March 2020, the World Health Organization (WHO) declared the COVID-19 infection a pandemic; from that moment on, the world was plunged into a state of unprecedented fear and uncertainty. The coronavirus disease, COVID-19, is a highly contagious respiratory disease caused by the severe acute respiratory syndrome coronavirus 2 (SARS-CoV-2).

Within only three months, the COVID-19 pandemic became the most severe global health challenge since the Spanish Flu one century ago [1]. The novel coronavirus has grown exponentially throughout the world, reaching globally over 117 million confirmed cases and 2.6 million deaths; in Italy, more than 3 million cases and 100 thousand deaths (status quo on 12 March 2021) [1,2].

As a primary defense emotion, fear plays an important role in adopting preventive behaviors and is used or even abused as a lever in communication, as it is thought that it can increase the effectiveness of a message. Fear is defined as a basic, intense emotion aroused by the detection of imminent threat, involving an immediate alarm reaction that mobilizes the organism by triggering a set of physiological changes. These include rapid heartbeat, redirection of blood flow away from the periphery toward the gut, tensing of the muscles, and a general mobilization of the organism to act [3]. Although there are differences with respect to anxiety, the terms are often used interchangeably in everyday language and our research does not aim to distinguish.

Multiple factors linked to culture, economy, ethics, health systems and environmental conditions play a role in the evolution of the pandemic. Individual and social dynamics interact in shaping the coping capacity of each community, and knowledge of psychological reactions is crucial for understanding the impact of COVID-19. It is also useful for designing and implementing appropriate initiatives to support coping strategies and psychological aspects related to the pandemic. Knowledge, education level and socioeconomic status (SES) can influence perception COVID-19 risk in a digital world [4]. Along with the epidemic, fear has been spreading and growing [5,6,7]. The global dimension of the present crises is unprecedent, and the negative impact is strongly influenced by personal and social emotions and behaviors typical of the “global risk society” [6].

The elements that characterize the risk perception of infectious diseases and the differences with non-communicable diseases must be fully understood in order to effectively manage risk communication and governance during a public health crisis. The links between fear and risk perception are multiple and largely inextricable, depending on social, cultural and contextual factors [8].

The elements that increase or mitigate fear can be classified into the following: voluntariness (if the risk is perceived as being voluntary it seems less dangerous); knowledge (a new risk evokes more fear); trust (faith in those who are managing the risk, such as public health institutions, makes the risk seem lower); and visibility (an invisible risk evokes more fear than a visible one) [9].

As the crisis develops, research has been progressing rapidly in a number of related areas such as the transmission modality, contagion risk, initial and specific symptoms and complications, the most effective treatment strategies, long-term effects and the preparation for the next pandemic wave. In a recent research study, a rapid COVID-19 screening based on self-reported symptoms was developed by a short diagnostic scale to detect subjects within a population, with specific symptoms potentially associated with COVID-19 [10].

While excessive fear hinders good management, insufficient fear can also play a negative role, leading individuals to ignore or reluctantly accept government measures to delay or prevent the spread of the virus or even facilitate reckless behaviors, oblivious to the risks they entail [8,11,12,13].

Other studies revealed that fear is an adaptive response in the presence of danger and can become chronic and burdensome when the threat is uncertain and continuous, as in the case of the Coronavirus disease (COVID-19) pandemic [14]. Fear is in any case a subjective conscious experience linked to numerous psychological and sociological factors [4,15,16].

The risk of contracting COVID-19 can increase with greater morbidity in the area, with crowding and mobility of the population, with less social distance, less access to health care and less education, which is linked to risky social behaviors [17]. The spread of the disease is influenced by people’s willingness to adopt preventative public health behaviors, linked to public risk perception. Personal experience with the virus, individualistic and prosocial values, hearing about the virus from friends and family, trust in government, science, and medical professionals, personal knowledge of government strategy, and personal and collective efficacy can be considered predictors of risk perception. Individualistic worldviews, personal experience, prosocial values, and social amplification through friends and family in particular are features that are related to risk perception and communication and to the adoption of preventative health behaviors [5]. Efforts to change behavior are critical in minimizing the spread of highly transmissible pandemics such as COVID-19, and the importance of risk perception in early interventions during large-scale pandemics is a crucial issue [18].

As fear may be a crucial construct in explaining individual and social behavior with reference to the COVID-19 pandemic, it is important to understand what people are exactly afraid of and which factors are triggering it. Therefore, it is essential to investigate the extent the fear relates to the individual her/himself or to people close to her/him, such as family members (FMs). The recommendations of “social distancing” have deeply influenced interpersonal relationships, and the physical distancing it entails not only protects, but also makes people who are physically close appear threatening. On the other hand, some people feel responsible for the good health of family and individuals in their community and for avoiding the spread of the virus. These behaviors can have profound consequences on relationships with significant others and may threaten an individual’s sense of security and the need to be a reference point for loved ones [19].

In light of these considerations, this study investigated how people in Italy were dealing with their risk perception and fears related to their own health as well as that of close FMs in the period of the COVID-19 pandemic. The research questions addressed are the following:

1. Which demographic and SES factors are linked to the fear of contracting the COVID-19 disease?
2. What role does self-perceived health play?
3. What role do chronic diseases, acute general symptoms, specific COVID-19-like symptoms or positive swab test results play?

The study’s main research goal was to analyze the participants’ fear of contagion for themselves and FMs, and to investigate the weight of different demographic, social and health status characteristics, including the presence of COVID-19-like symptoms and positive SARS-CoV-2 swab test, in association with fear.

Verifying these elements should allow us to understand the specificity of the situation in Italy, which has never experienced a phenomenon like the one in progress. The results were discussed in the light of recent scientific literature referring to other countries affected by the pandemic to obtain take home messages for pandemic governance and decision making.

The differences between fear for oneself and for FMs were discussed in the light of the achieved results.

## 2. Methods

### 2.1. Study Design and Setting

The present study is based on EPICOVID19 (https://epicovid19.itb.cnr.it/, accessed on 19 March 2021), an extensive national research study carried out in 2020 during the peak of the pandemic in Italy. EPICOVID19 is a national internet-based, cross-sectional survey led by a multidisciplinary research team operating in three biomedical Institutes of the National Research Council, in the Sacco Hospital, University of Milan, the Italian Society of Geriatrics and Gerontology (SIGG) and the Italian Society of Infectious and Tropical Diseases (SIMIT) [10,20,21,22]. The survey was launched on 13 April 2020 and data were collected until 2 June 2020; it targeted adult volunteers living in Italy during the first lockdown period set by the Italian Government (from 9 March to 18 May 2020) in response to the growing pandemic of COVID-19 in the country. The survey began approximately one month after the start of the first lockdown due to the necessary trigger time and ran for a reasonable time after its end, having reached 200,000 responses and in the presence of a declining epidemic curve. The study was registered in the international repository for clinical and epidemiological investigations (ClinicalTrials.gov NCT04471701). EPICOVID19 is still an active study, investigating new aspects of the health emergency, in particular the ongoing vaccination campaign and the performance of serological tests and molecular swabs.

### 2.2. Recruitment

To encourage participation in the EPICOVID19 study, the link to the online survey was shared using social media (Facebook, Twitter, Instagram and WhatsApp), press releases, web pages, local radio and television stations and institutional channels. The inclusion criteria were age >18 years; access to a mobile phone, computer, or tablet with internet connectivity; and provision of web-based consent to participate in the study.

### 2.3. Development of the Web-Based Questionnaire

EPICOVID19 was developed by the working group after a literature review of existing research into COVID-19 and already available standard and validated instruments, as described in detail elsewhere [10,20,21,22].

The questionnaire was adapted to the national context and implemented using the European Commission’s open-source official EU Survey management tool (https://ec.europa.eu/eusurvey/, accessed on 12 February 2021). The participants were asked to complete the self-administered 38-item questionnaire, which mainly contained mandatory and closed questions divided into six sections: (1) demographic and SES data; (2) clinical evaluation; (3) personal characteristics and health status; (4) housing conditions; (5) lifestyle; (6) behaviors following the first lockdown period [20] (see the Supplementary Materials).

### 2.4. Data Collection and Variables

The demographic and SES information included sex (men and women), age (categorized as 18 to 39, 40 to 59 and ≥60 years), educational level (primary school or less, middle or high school and university degree or postgraduate degree), and occupational status (unemployed, employed, retired, student and other). The COVID-19 related symptoms included fever >37.5 °C for at least three consecutive days; headache, chest pain, myalgia, olfactory and taste disorders, shortness of breath, and heart palpitations; gastrointestinal disturbances, including nausea, vomiting and diarrhea; conjunctivitis; and sore throat, rhinorrhea, and cough (all dichotomized as present/absent). The self-reported chronic conditions investigated were the diseases of heart, lung, kidney, liver, immune system, metabolism, hypertension, tumors, depression and/or anxiety. Self-perceived health status had five possible answers: very bad, bad, adequate, good and very good.

The month of onset of the first symptoms (February/March/April 2020), nasopharyngeal swab (NPS) test results (categorized as not performed, performed with a negative result, performed with a positive result and performed with an unknown result), flu and pneumococcal vaccinations in 2019, contact with COVID-19 confirmed or suspected individuals were collected and included in the analysis. A heterogeneity evaluation among results in the 20 Italian regions was also carried out.

### 2.5. Outcome Variable

To evaluate the association with the selected variables of the EPICOVID19 questionnaire, the dichotomous classification of perceived fear of COVID-19 contagion was considered: None or Low level vs. Medium or High level of fear. The specific questions about the experience of fear were the following: “Do you fear getting infected with the coronavirus (COVID-19)?” and “Do you fear your family being infected with the coronavirus (COVID-19)?” with the possible answers formulated as follows: No; Just a little bit; Neutral; Quite enough; Yes, a lot.

### 2.6. Statistical Analysis

The descriptive analyses were carried out using t tests for continuous variables (age, number of symptoms, number of diseases), Chi-square tests for categorical variables (sex, education, occupation, health, contacts, swab, vaccines, symptoms and diseases) to evaluate whether the level of fear of contagion differed by the abovementioned variables. The multivariate analysis carried out through logistic regression provided results on the association between the level of fear and the variables above, expressed as adjusted Odds Ratios (aORs), with 95% Confidence Intervals (CIs). All the analyses, adjusted for sex, age, educational level and occupational status, were performed using Stata 15.0 version (StataCorp LP, College station, Texas, USA) and a two-sided *p*-value < 0.05 was considered statistically significant.

### 2.7. Ethics and Consent Form

The Ethics Committee of the National Institute Infectious Diseases I.R.C.C.S. Lazzaro Spallanzani, Italy (Protocol No. 70, 4 December 2020) approved the EPICOVID19 study protocol. When participants first accessed the web-based platform, they were informed about the purpose of the study, the data to be collected, and the methods of data storage before filling in the informed consent form. The planning, conduction and reporting of the studies was in line with the Declaration of Helsinki, as revised in 2013. Data were handled and stored in accordance with the European Union General Data Protection Regulation (EU GDPR) 2016/679, and data transfer was safeguarded by encrypting/decrypting and password protection.

## 3. Results

### 3.1. The Fear of Contagion for Individuals and for Family Members

Out of 207,341 persons participating in the initiative, 198,822 provided the consent to participate and fully responded to the questionnaire items (95.9%), and of them 60% were women, with an average age of 47.3 years (SD 14.3) vs. 48.9 years (SD 15.1) among men. The answers according to the graduation of perceived fear of COVID-19 contagion were: No (none, *n* = 47,360; 24%), Low (neutral and little, *n* = 80,696; 40%), Medium-High (enough and much, *n* = 70,772; 36%). Fear for FMs was distributed as follows: No (*n* = 16,305; 8%), Low (*n* = 51,700; 26%); Medium-High (*n* = 130,823; 66%). To evaluate the association with the selected variables of the questionnaire, the dichotomous classification of fear was considered: classes No and Low (64% fear for themselves and 34% fear for FMs) against Medium-High (36% and 66%, respectively) (Table 1).

### 3.2. Demographic and SES Factors Linked to the Fear of Contracting COVID-19

Fear prevailed when referring to FMs over fear for oneself (66% vs. 36%); 70% and 39%, respectively, among women compared to 59% and 31% among men (Table 1). The gender gap was higher for fear for FMs (aOR = 0.61; 0.59–0.62) than for fear for themselves (aOR = 0.68; 0.67–0.69). Fear for oneself had greater representation in the age group above 40 years (38.3%) and increased even further above 60 years (39.1%) compared to subjects aged 18–39 years (29.1%). Instead, fear for FMs was much more represented in all age groups, decreasing from the youngest class (72.5%), to the intermediate class (65.9%), to the over 60s (56.9%) (Table 1). The average age of those who were afraid for themselves was significantly higher (49.7 years) than those who were not afraid (46.9 years) (t-test =40.2; *p* < 0.001), while those who were afraid for their FMs were on average younger than those who were not afraid (46.7 versus 50.5 years; t-test = 54.7; *p* < 0.001) (Data not shown). The fear for oneself was lower in those with a higher level of education, while no differences by education were observed for fear for FMs. The proportion of individuals with fear for themselves was higher among the retirees (39.5%) and lower among students (21.8%) who showed a high percentage of fear for FMs (69.5%), which was instead higher among the unemployed (70.7%) (Table 1).

### 3.3. Self-Perceived Health and Fear

Fear for oneself had greater representation among the small group of subjects who perceived themselves to be in poor health (53.9%) than those who declared themselves to be neutral (47.9%), and even more among the majority of subjects with good to very good health (33.2%). Fear for FMs was high in those reporting very bad or bad (71.7%) and intermediate (70.7%) health status, and lower in those with a good or very good health status (64.9%) (Table 1).

### 3.4. Fear and Vaccinations

In the analyzed EPICOVID19 dataset, 21% of respondents reported flu vaccination and 3.6% reported pneumococcal vaccination in the previous 2019 year. Among those who reported flu vaccination, 40.8% were frightened by the possibility of COVID-19 contagion for themselves compared to 34.2% who were not vaccinated (aOR = 1.29; 1.26–1.32). Similar results were obtained for the pneumococcal vaccination, with 39.8% fearful among the vaccinated versus 35.4% among the unvaccinated ((aOR = 1.21; 1.15–1.27) data not shown)). When fear for FMs was considered, the aORs for flu and pneumococcal vaccination were 1.30 (1.27–1.33) and 1.09 (1.03–1.14), respectively (Table 1). Flu-vaccinated respondents reported most often chronic diseases: heart disease (aOR = 4.4), hypertension, lung, kidney, metabolic diseases, tumors (aORs > 2 < 3), immunologic or liver diseases, depression and/or anxiety (aORs > 1 < 2) and allergies (aOR = 0.9). Similar results emerged considering subjects who had undergone the pneumococcal vaccination in 2019.

### 3.5. Fear and Contact with People

Fear of contagion for oneself was reported by 41.4% of respondents who had contact with persons who tested positive for COVID-19 compared with 35.1% among those without contacts, aOR = 1.31 (1.26–1.35). A higher odds ratio was observed when considering fear for FMs, aOR = 1.45 (1.39–1.50). The proportions of scared individuals decreased in the case of contact with people suspected of being positive versus people without contacts: 36.7% versus 33.3% with an aOR = 1.19 (1.17–1.22). Stronger associations emerged when fear for FMs was investigated: 69.3% of respondents who had contact with persons with confirmed COVID-19 infections compared with 60.2% among people with no contact, adjusted aOR = 1.44 (1.39–1.50). People who ignored whether they had contacts with infected individuals had higher odds of fear both for themselves (aOR = 1.41; 1.37–1.46) and for FMs (aOR = 1.81; 1.75–1.87) (Table 1).

### 3.6. Fear and Nasopharyngeal Swab (NPS)

Individuals declaring fear for themselves was 35.3% among those who had not performed the NPS test (no NPS), 42.3% among those with negative NPS and 49.8% among those with positive NPS. aORs were 1.33 (1.26–1.41) for negative NPS compared with the No NPS group and 1.70 (1.54–1.87) for positive NPS versus No NPS. Similar differences emerged considering the fear for FMs: 65.5% for the No NPS group, 73.1% among NPS with a negative result and 75.8% among positive NPS; the aORs for negative NPS and positive NPS compared with No NPS were 1.35 (1.27–1.44) and 1.79 (1.59–2.00), respectively. Even for individuals waiting for the test results, fear of contagion was still high, considering fear both for themselves (aOR = 1.51; 1.28–1.76) and for FMs (aOR = 1.57; 1.31–1.89) (Table 1).

### 3.7. Fear, COVID-19-Like Symptoms and Chronic Diseases

Among the respondents who declared at least one COVID-19-like symptom, 18% were those with fear for themselves rather than those without fear (aOR = 1.18); the excess rose to 34% when considering fear for FMs (aOR = 1.34). When the number of self-reported symptoms was between two and five, the aOR was 1.2 for fear for self and 1.3 for fear for FMs; above five reported symptoms, the excess of fear increased to 1.9 and 1.5, respectively (Table 2). For all symptoms investigated, both the fear for oneself and fear for FMs was significantly higher than for people declaring no symptoms. Fear for oneself was higher when associated with chest pain, olfactory/taste disorders, heart palpitations, (all with aOR > 1.5), shortness of breath (aOR = 1.5) and fever (aOR = 1.4). For the same symptoms, fear for FMs had similar excesses, with the addition of gastrointestinal disorders and myalgia (aOR = 1.3 and 1.4, respectively); for conjunctivitis, sore throat/rhinorrhea, cough and headache, the aORs were lower than 1.2. (Table 2).

When at least one of the nine chronic diseases investigated by the EPICOVID19 questionnaire was declared, a positive association with fear for oneself compared with no fear was observed (aOR = 1.3). Fear increased when one, two or more than two diseases were declared (aORs = 1.2, 1.5, 1.7, respectively) (Table 3). The association with fear was significantly higher for lung disease, kidney disease and depression and/or anxiety (all with OR = 1.3–1.4), with a statistically significant excess also for the other diseases investigated (aORs = 1.15–1.24). Fear for FMs showed similar but slightly lower aORs for each chronic disease. The analysis of fear for FMs confirmed the excesses found when considering fear for oneself, although with 5–15% lower aORs, except for immunological diseases and depression and/or anxiety which showed similar excesses (Table 3).

### 3.8. Fear and Region of Residence

A large number of participants were reached throughout Italy with a coverage proportional to the distribution of COVID-19 infection over the same time period, indicating that residents in more affected areas (as three large Northern regions) were more inclined and attracted to participate in the survey. In a previous article on the results of the EPICOVID survey, a high correlation between regional COVID-19 incidence data and the response rate to questionnaires was reported [20]. It is reasonable to assume that a systematic error due to self-selection of individuals who were more worried and prone to fear had an increasing impact in southern regions compared to central and northern regions, as they were characterized by increasing rates of survey participants. In addition, the fact that in adjusting for some variables there remains a pattern of inverse correlation between the 20 regions with the proportion of individuals with COVID-19 infection and statements of fear supports the hypothesis that the motivations are likely inherent cultural and social aspects and not included in the questionnaire. With regard to living with the elderly or immunocompromised or chronically ill, it is noteworthy that all the sensitivity analyses carried out led to similar results as the analyses without adjusting for this variable, even with a reduction of the adjusted estimates. Regarding the differences in fear observed between regions, although one might reasonably expect that living with frail people plays an important role and might partially explain these differences, the pattern does not substantially change, pointing to other explanatory factors not considered in the questionnaire (data not shown).

## 4. Discussion

### 4.1. The Fear of Contagion for Individuals and for FMs

The present study was based on the data of the anonymous, self-administered web-based EPICOVID19 survey [10,20,21,22] completed by a large number of adults residing in Italy during the first lockdown period due to the SARS-CoV-2 infection established by the Italian government between 9 March and 4 May 2020. This work was aiming to investigate how people were reacting to the possibility of being infected by COVID-19 or of seeing loved ones infected, their perception of the risk of contagion and their own self-perceived health.

The response rate to the questionnaire was high, with 95.9% out of 207,341 persons participating in the initiative. Our study found that more than one-third EPICOVID19 participants reported fear of contagion for themselves, and two-thirds reported fear for FMs. The analysis showed that sex, age, education and occupation were significantly associated with the perception of fear, together with factors related to health, habits and behavior.

### 4.2. Demographic and SES Factors Linked to the Fear of Contracting COVID-19

There was a significant remarkable difference in the prevalence of fear between men and women; a higher fear level prevailed among women. Although men are hit harder by COVID-19 than women [23], previous studies indicated that women are more worried about the spread of the infection. In a recent Cuban population study, being female was a predictor of medium and high levels of fear of COVID-19 [24], and also in several other researches it emerged that being female is significantly associated with a greater psychological impact of the outbreak and higher levels of stress, anxiety [25], insomnia, perceived stress, adjustment disorders and depression [26,27]. Galasso et al. (2020), in a study carried out in eight countries, showed that women are more likely to perceive COVID-19 as a very serious health problem and to agree and comply with restraining public policy measures [28]. The present study’s findings confirmed the results of previous studies performed in other countries, observing that fear of COVID-19 contagion was a psychological distress particularly felt by women.

Fear for oneself was found to increase with age and decrease with rising educational levels. It is possible to relate the increase of education level to a better understanding of the multiple aspects underlying the pandemic situation and to a growing self-efficacy, which is the individual’s belief in her/his own ability to complete complex tasks and assume responsibility; self-efficacy consciousness affects people’s actions, expectations and perceptions, being even more important than actual abilities and skills in terms of explaining individual differences [29]. Self-efficacy is closely linked to agency, the ability to act and find a space in society, generating awareness of physical behavior and attitudes and control over emotions, including fear [30,31].

With regard to employment status, three categories revealed quite different attitudes. The highest level of fear for themselves was observed for retired people (39.5%), students were three times more afraid for FMs (69.5%) than for themselves (21.8%), whereas workers were at a similar level to the overall sample (Table 1). This can be explained, at least in part, considering the different agency of the three typologies, confirming the results explained above about education and the fact that it was common knowledge during the first lockdown that the average age of death for someone with Covid-19 was over 70, with a call for collective efforts to protect the elderly.

In research carried out in Hong Kong on survey data, more respondents with higher than lower socioeconomic scores (SES) reported perceived benefits on family physical and mental health and family relationships, but more respondents with lower SES reported perceived harm on family income [32]. Interventions aimed at low income and less educated adults should be developed to improve preventive behaviors in this vulnerable group [33].

Approximately 500,000 people died worldwide within the first six months of the COVID-19 pandemic. The virus itself, as well as the related political decisions, intensified an increasing feeling of fear in billions of people. Overall, as the pandemic escalated, fear of being infected increased significantly. According to a recent study by a German psychology research group, the strongest predictors of fear were personality variables, as well as education, sex and being an at-risk person [34].

### 4.3. Self-Perceived Health and Fear

The self-perceived state of health indicates people’s general perception of their health and includes both the physical and psychological dimensions; in the present study, self-assessed good health associated with a low level of fear of COVID-19 contagion was found. Respondents who reported worse health had the highest level of fear for themselves and their loved ones. This supports the hypothesis of a direct relationship between self-perceived vulnerability to infection and increased fear of getting infected. In this regard, perceived health status may be a useful indicator of health care needs and a highly sensitive dimension to social factors, as well as a measure associated with actual health status and demand for health care [35,36].

### 4.4. Fear and Vaccinations

Vaccination is one of the most cost-effective ways of avoiding disease—it currently prevents 2–3 million deaths a year, and a further 1.5 million could be avoided if global coverage of vaccinations improved [37]. Perceived threat acts as a motivational factor to perform behaviors that facilitate disease prevention; the attitude towards vaccination is a coping strategy that is determined by knowledge, prior conditions, health care advice and also by fear. Given the possible coexistence of influenza, bacterial infections and COVID-19 [21], vaccination should be considered a crucial preventive measure. The 21% of respondents who declared they had undergone a flu vaccination in 2019 can be considered individuals with a positive preventive attitude. Consistent results emerged when considering anti-pneumococcal vaccination. Noale et al. found that anti-pneumococcal and flu vaccinations were associated with a decreased probability of a COVID-19 NPS positive test in younger participants and that a significantly lower probability of a positive test result was detected in individuals ≥65 years who received an anti-pneumococcal vaccination [21]. The results presented demonstrate more fear of being infected in vaccinated people, both for themselves and for FMs, confirming the possible role of fear in promoting preventive attitudes. Our survey was related to the very first surge of the pandemic. In 2019 the possibility of vaccine hesitancy was anticipated by the WHO. Vaccine hesitancy threatens to reverse progress made in tackling vaccine-preventable diseases. The reasons why people choose not to vaccinate are complex: complacency, inconvenience in accessing vaccines and lack of confidence are key reasons underlying hesitancy. Health community workers can be considered the most trusted influencer of vaccination decisions [37].

### 4.5. Fear and Contact with People

Analysis of the responses revealed that contact with confirmed persons infected with COVID-19 resulted in greater fear for FMs than for oneself, which increased further in the case of contact with suspected COVID-19 cases. When people with suspected contact are compared with people without suspected contact, more fear is confirmed for the first condition, both for themselves and for FMs. Doubtful contact is likely to cause greater anxiety and difficulty in coping, both of which evoke fear, than contact with people with a verified infection, in which case established guidelines can be followed. These uncertainties had direct implications for the daily life and mental health of the population, with a major generalized psychosocial impact due to the high infectivity and mortality rate of the disease. Doubts about virus transmission and uncertainty about infecting family and friends led to insecurity and fear. Insufficient control measures (and a lack of effective therapeutic measures) exacerbated the situation and may have increased mental health problems [38,39].

### 4.6. Fear and Nasopharyngeal Swab (NPS)

When we look at people who have undergone an NPS test, we see that in the case of a positive result fear increases significantly, as can be expected, particularly when one considers the fear for FMs. People who are waiting for an answer to the test also show a reaction of fear. This kind of response should be considered by health officials who are planning testing campaigns for the population and in general when preparing awareness-raising campaigns.

### 4.7. Fear, COVID-19-Like Symptoms and Chronic Diseases

Analyzing the association between fear and COVID-like symptoms, there was a constant increase in reported fear of contagion, starting from one and going up to more than five self-reported symptoms. This occurred both out of fear for themselves and for FMs. The multivariate analysis showed that the most informative symptoms with respect to perceived fear were chest pain, loss of taste/smell, heart palpitations and shortness of breath, which have a stronger association with COVID-19 [10,20]. Conversely, those that generated the least fear were conjunctivitis, sore throat/rhinorrhea, cough and headache. Cough, fever, asthenia, myalgia and alterations in smell or taste have been repeatedly reported as the most frequent clinical symptoms at the onset of the COVID-19 disease [40,41,42], the same reported as reliable indicators of COVID-19 infection by the EPICOVID19 web survey [20]. We also know that respiratory problems can mostly occur between one week and 10 days after the onset of symptoms [10,43,44,45,46,47,48]. Concerning COVID-like symptoms, Bastiani et al. evaluated the capability of self-reported symptoms in discriminating COVID-19 to identify individuals who needed to undergo instrumental measurements through the definition of a validated short scale (EPICOVID19 Diagnostic Scale) [10].

An increase in the percentage of individuals reporting fear emerged as the number of illnesses reported increased beyond one. Some illnesses were found to be more strongly associated with fear of contagion, specifically lung disease, kidney disease, hypertension and depression and/or anxiety. A review of published/preprint articles reported that diabetes, hypertension and cholesterol levels possess an apparent relation to COVID-19 severity [49]. Other comorbidities, such as cancer, and in particular lung cancer [50], kidney disease and stroke, must be further evaluated to determine the strength of relationship with COVID-19 infection [49].

As expected, depression and/or anxiety was also one of the most fear-related factors. There are several psychological vulnerability factors [51] linked to depression and/or anxiety [52,53,54]. Anxiety may also play an important role in the fear and depression duet, since it is defined by a repetitive, negative and catastrophic thought process [55]. Such thoughts could be related to health concerns and uncertainty and may be relevant to oneself or to loved ones [56]. More concern, and fear, would be expected if an individual perceived a more personal threat (e.g., due to worse overall health) or a specific threat to loved ones (e.g., grandparents).

A similar pattern that emerged when considering fear for FMs reinforces the importance of considering the presence, number and type of chronic illnesses in order to give targeted recommendations, in particular to motivate individual and social distancing measures.

### 4.8. Fear and Region of Residence

A large number of participants were reached throughout Italy with a coverage proportional to the distribution of the SARS-CoV-2 infection over the same time period, indicating that residents in more affected areas (three large Northern regions) were more inclined and attracted to participate to the survey. In a previous article on the results of the EPICOVID survey, a high correlation between regional COVID-19 incidence data and the response rate to questionnaires was reported [20]. It is reasonable to assume a systematic error due to self-selection of more worried/fear-prone subjects and more representation in the southern regions than in the central and northern regions where a higher rate of participants was recorded. Furthermore, the fact that by adjusting for several variables an inverse correlation model between regions with proportion of subjects with COVID-19 and fear statement remains makes it reasonable to assume that further cultural and social factors should be considered.

With regard to living with elderly or immunocompromised or chronically ill persons, it should be noted that all analyses adjusted for this variable gave similar results to those without adjustment. As for the differences in fear observed between regions, although as one might reasonably expect that living with frail persons plays an important role, the pattern did not substantially change, reinforcing the hypothesis of other explanatory factors.

### 4.9. Limitations and Strengths of the Study

The present study is based on a cross-sectional Italian survey (EPICOVID19) and it is therefore unable to determine the mechanism or causal ordering of effects. Moreover, the data are mainly based on self-reported measures, which have some limitations due to their potential for social desirability and recall biases. The sample was obtained through recruitment based on voluntary participation, so it is subject to selection bias. Given the voluntary nature of the on-line survey, it did not intend to assess a representative sample of the general population. Nevertheless, the extensive participation allowed for evaluating a convenience sample that was quite balanced, although it shifted toward women and younger respondents with a higher level of education and health-consciousness [20]. However, EPICOVID19, having collected questionnaires from almost 200.000 respondents, is the largest Italian web-based survey on COVID-19 symptoms carried out during the 2020 spring peak of the epidemic in Italy.

## 5. Conclusions

It is well known that fear and the connected risk perception directly and indirectly plays a role in the preventive behaviors that individuals adopt and the interventions they agree with. Studying fear is also important because of its links with people’s health conditions, considering the protective significance of fear as personal and collective assumption of responsibility in the face of an uncertain future [57,58].

The present research, developed during the first outbreak of the COVID-19 pandemic in Italy, suggested an overall acknowledgement of responsibility in the population which showed that participants were more worried about FMs than for themselves. Fear was higher among women than men and decreased with higher levels of education (fear for oneself was lower in those with higher levels of education but no differences by education were observed for fear for FMs) and in those who perceived themselves as having good health.

Some results show that uncertainty is an important determinant of fear, in particular it is interesting that unawareness of having had contact with people suspected of being infected is a greater source of fear than known contacts; similarly, those who were waiting for a response to the nasopharyngeal swab had an intermediate fear between negative and positive responses. The finding that people previously vaccinated for influenza or pneumococcal disease were more afraid than the unvaccinated is an indication that frailty prevails as a higher risk compared to vaccination protection.

Fear increased when one or more chronic diseases and symptoms were declared, and in particular those recognized as being associated with COVID-19.

The results of this study, in agreement with other authors, showed a link between fear and depression/anxiety which confirms the importance of taking mental health needs into account, and this may also be particularly relevant when using a personalized approach to reduce health inequalities [57,58].

In addition to demographic and SES characteristics, the positive association between health status and the fear of becoming infected should be taken into serious consideration when protective measures for the most vulnerable people are being designed and implemented.

The knowledge gained form these results should be used to produce tailored messages and shared public health decisions.

Special caution should be applied in communicating about the COVID-19 pandemic to avoid using fear as a lever of emotions instead of rationality as a tool to impose behaviors and decisions. Even when managing fear, communication should improve trust between institution and citizens, promoting involvement and collaboration.

## Figures and Tables

**Table 1:** Fear for themselves and for family members (FMs) according to selected variables obtained by the self-administered EPICOVID19 survey questionnaire.

Variable	Category	Fear for Themselves				Chi-Squared	*p*	Fear for FMs				Chi-Squared	*p*
		No		Yes				No		Yes			
		N.	*%*	N.	*%*			N.	*%*	N.	*%*		
All		128,056	*64.4*	70,772	*35.6*			68,005	*34.2*	130,823	*65.8*		
		N.	*%*	N.	*%*			N.	*%*	N.	*%*		
**Sex**	**Female**	72,304	*60.9*	46,357	*39.1*			35,212	*29.7*	83,449	*70.3*		
	**Male**	55,752	*69.5*	24,415	*30.5*	1547.7	<0.0001	32,793	*40.9*	47,374	*59.1*	2681.8	<0.0001
**Age**	**<40 years**	44,341	*70.9*	18,241	*29.1*			17,223	*27.5*	*45,359*	*72.5*		
	**40–59**	54,761	*61.7*	34,012	*38.3*			30,309	*34.1*	58,464	*65.9*		
	**≥60**	28,954	*61.0*	18,519	*39.1*	1662	<0.0001	20,473	*43.1*	27,000	*56.9*	2921	<0.0001
**Educational level**	**No/low**	6621	*55.8*	5239	*44.2*			3835	*32.3*	8025	*67.7*		
	**Intermediate**	42,524	*61.8*	26,276	*38.2*			23,312	*33.9*	45,488	*66.1*		
	**Graduate/post-graduate**	78,911	*66.8*	39,257	*33.2*	873.5	<0.0001	40,858	*34.6*	77,310	*65.4*	28.8	<0.0001
**Occupation**	**Worker**	87,748	*64.1*	49,108	*35.9*			44,912	*32.8*	91,944	*67.2*		
	**Student**	10,503	*78.2*	2933	*21.8*			4095	*30.5*	9341	*69.5*		
	**Unemployed**	5715	*62.2*	3469	*37.8*			2690	*29.3*	6494	*70.7*		
	**Retired**	17,126	*60.5*	11,186	*39.5*			12,236	*43.2*	16,076	*56.8*		
	**Other**	6964	*63.1*	4076	*36.9*	1419.4	<0.0001	4072	*36.9*	6968	*63.1*	1325.2	<0.0001
**Self-perceived health**	**Poor-very poor**	835	*46.1*	976	*53.9*			512	*28.3*	1299	*71.7*		
	**Intermediate**	15,488	*52.1*	14,257	*47.9*			8724	*29.3*	21,021	*70.7*		
	**Good-very good**	111,733	*66.8*	55,539	*33.2*	2656	<0.0001	58,769	*35.1*	108,503	*64.9*	407	<0.0001
		**N.**	***%***	**N.**	***%***	**Odds Ratio §**	**95% CI**	**N.**	***%***	**N.**	***%***	**Odds Ratio §**	**95% CI**
**Contact with infected**	**No**	118,66	*64.9*	64,143	*35.1*	1.00		63,789	*34.9*	119,014	*65.1*	1.00	
	**Yes**	9396	*58.6*	6629	*41.4*	1.31	1.26–1.35	4216	*26.3*	11,809	*73.7*	1.45	1.39–1.50
**Contact with suspected**	**No**	59,155	*66.7*	29,590	*33.3*	1.00		35,337	*39.8*	53,408	*60.2*	1.00	
	**Yes**	54,911	*63.3*	31,825	*36.7*	1.19	1.17–1.22	26,576	*30.7*	5998	*69.3*	1.44	1.41–1.47
	**Unknown**	13.99	*59.9*	9357	*40.1*	1.41	1.37–1.46	5912	*25.3*	17,435	*74.7*	1.81	1.75–1.87
**Nasopharyngeal swab**	**No**	123,975	*64.7*	67,539	*35.3*	1.00		66,100	*34.5*	125,414	*65.5*	1.00	
	**Yes, negative**	2889	*57.7*	2115	*42.3*	1.33	1.26–1.41	1346	*26.9*	3658	*73.1*	1.35	1.27–1.44
	**Yes, positive**	842	*50.2*	834	*49.8*	1.73	1.54–1.87	406	*24.2*	1270	*75.8*	1.79	1.59–2.00
	**Waiting for result**	350	*55.2*	284	*44.8*	1.54	1.28–1.76	153	*24.1*	481	*75.9*	1.57	1.31–1.89
**Flu vaccine**	**No**	103,302	*65.8*	53,706	*34.2*	1.00		5377	*34.2*	103,238	*65.8*	1.00	
	**Yes**	24,754	*59.2*	17,066	*40.8*	1.29	1.26–1.32	14,235	*34.0*	27,585	*66.0*	1.30	1.27–1.33
**Pneumococcal vaccine**	**No**	123,735	*64.6*	67,915	*35.4*	1.00		65,369	*34.1*	126,281	*65.9*	1.00	
	**Yes**	4321	*60.2*	2857	*39.8*	1.21	1.15–1.27	2636	*36.7*	4542	*63.3*	1.09	1.03–1.14

§ Adjusted by sex, age, educational level, occupation.

**Table 2:** Fear for themselves and for FMs according to symptoms obtained by the self-administered EPICOVID19 survey questionnaire.

Variable	Category	Fear for Themselves				*t*-Test	*p*	Fear for FMs				*t*-Test	*p*
		No		Yes				No		Yes			
		N.	Mean (SD)	N.	Mean (SD)			N.	Mean (SD)	N.	Mean (SD)		
Symptoms		128,056	1.49 (1.80)	70,772	2.08	32.7	<0.0001	68,005	1.31 (1.71)	130,823	1.76 (1.99)	52.8	<0.0001
		N.	*%*	N.	*%*	Odds Ratio §	95% CI	N.	*%*	N.	*%*	Odds Ratio §	95% CI
	**No**	52,194	*66.3*	26,541	*33.7*	1.00		31,050	*39.4*	47,685	*60.6*	1.00	
	**Yes**	75,862	*63.2*	44,231	*36.8*	1.18	1.15–1.21	36,955	*30.8*	83,138	*69.2*	1.34	1.31–1.36
	**No**	52,194	*66.3*	26,541	*33.7*	1.00		31,050	*39.4*	47,685	*60.6*	1.00	
	**1**	26,248	*66.8*	13,074	*33.3*	1.01	0.98–1.03	14,047	*35.7*	25,275	*64.3*	1.15	1.13–1.17
	**2–5**	44,401	*62.9*	26,210	*37.1*	1.21	1.18–1.23	20,617	*29.2*	49,994	*70.8*	1.29	1.25–1.33
	**>5**	5213	*51.3*	4947	*48.7*	1.90	1.83–1.99	2291	*22.6*	7869	*77.4*	1.54	1.47–1.61
**Fever**	**No**	118,303	*65.0*	63,692	*35.0*	1.00		63,289	*34.8*	118,706	*65.2*	1.00	
	**Yes**	9753	*57.9*	7080	*42.1*	1.38	1.33–1.43	4,716	*28.0*	12,117	*72.0*	1.31	1.26–1.35
**Headache**	**No**	93,854	*65.3*	49,794	*34.7*	1.00		52,929	*36.9*	90,719	*63.2*	1.00	
	**Yes**	34,202	*62.0*	20,978	*38.0*	1.19	1.17–1.22	15,076	*27.3*	40,104	*72.7*	1.33	1.30–1.36
**Myalgia**	**No**	104,710	*65.7*	54,656	*34.3*	1.00		57,070	*35.8*	102,296	*64.2*	1.00	
	**Yes**	23,346	*59.2*	16,116	*40.8*	1.28	1.25–1.31	10,935	*27.7*	28,527	*72.3*	1.38	1.35–1.41
**Olfactory/taste disorders**	**No**	122,020	*65.0*	65,713	*35.0*	1.00		65,280	*34.8*	122,453	*65.2*	1.00	
	**Yes**	6036	*54.4*	5059	*45.6*	1.54	1.48–1.59	2725	*24.6*	8370	*75.4*	1.52	1.45–1.59
**Shortness of breath**	**No**	121,945	*64.9*	65,975	*35.1*	1.00		65,145	*34.7*	122,775	*65.3*	1.00	
	**Yes**	6111	*56.0*	4797	*44.0*	1.48	1.43–1.55	2860	*26.2*	8048	*73.8*	1.42	1.35–1.48
**Chest pain**	**No**	120,739	*65.1*	64,854	*34.9*	1.00		64,670	*34.9*	120,923	*65.1*	1.00	
	**Yes**	7317	*55.29*	5918	*44.7*	1.55	1.49–1.61	3335	*25.2*	9900	*74.8*	1.48	1.42–1.54
**Heart palpitations**	**No**	121,319	*65.1*	64,946	*34.9*	1.00		65,123	*35.0*	121,142	*65.0*	1.00	
	**Yes**	6737	*53.6*	5826	*46.4*	1.54	1.49–1.60	2882	*22.9*	9681	*77.1*	1.62	1.55–1.69
**Gastrointestinal disturbances**	**No**	108,105	*65.3*	57,447	*34.7*	1.00		59,125	*35.7*	106,427	*64.3*	1.00	
	**Yes**	19,951	*60.0*	13,325	*40.0*	1.29	1.25–1.32	8880	*26.7*	24,396	*73.3*	1.39	1.36–1.43
**Conjunctivitis**	**No**	116,619	*64.8*	63,306	*35.2*	1.00		62,206	*34.6*	117,719	*65.4*	1.00	
	**Yes**	11,437	*60.5*	7466	*39.5*	1.16	1.13–1.19	5799	*30.7*	13,104	*69.3*	1.22	1.18–1.26
**Sore throat/rhinorrhea**	**No**	87,407	*65.3*	46,462	*34.7*	1.00		48,964	*36.6*	84,905	*63.4*	1.00	
	**Yes**	40,649	*62.6*	24,310	*37.4*	1.18	1.16–1.21	19,041	*29.3*	45,918	*70.7*	1.27	1.24–1.29
**Cough**	**No**	102,052	*65.2*	54,595	*34.8*	1.00		55,460	*35.4*	101,187	*64.4*	1.00	
	**Yes**	26,004	*61.6*	16,177	*38.4*	1.19	1.17–1.22	12,545	*29.7*	29,636	*70.3*	1.24	1.21–1.27

§ Adjusted by sex, age, educational level, occupation.

**Table 3:** Fear for themselves and for FMs according to chronic diseases by the EPICOVID19 survey questionnaire.

Variable	Category	Fear for Themselves				*t*-Test	*p*	Fear for FMs				*t*-Test	*p*
		No		Yes				No		Yes			
		N.	Mean (SD)	N.	Mean (SD)			N.	Mean (SD)	N.	Mean (SD)		
Diseases		128,056	0.43 (0.72)	70,772	0.58 (0.83)	40.0	<0.0001	68,005	0.48 (0.75)	130,823	0.49 (0.77)	3.3	<0.0001
		N.	*%*	N.	*%*	Odds Ratio §	95% CI	N.	*%*	N.	*%*	Odds Ratio §	95% CI
	**No**	85,839	*67.5*	41,418	*32.5*	1.00		43,735	*34.4*	83,522	*65.6*	1.00	
	**Yes**	42,217	*59.0*	29,354	*41.0*	1.31	1.28–1.34	24,270	*33.9*	47,301	*66.1*	1.24	1.22–1.27
	**No**	85,839	*67.5*	41,418	*32.5*	1.00		43,735	*34.4*	83,522	*65.6*	1.00	
	**1**	31,835	*60.8*	20,532	*39.2*	1.24	1.21–1.27	17,863	*34.1*	34,504	*65.9*	1.19	1.16–1.22
	**2**	8028	*55.2*	6511	*44.8*	1.49	1.44–1.55	4912	*33.8*	9627	*66.2*	1.38	1.33–1.44
	**>2**	2354	*50.5*	2311	*49.5*	1.73	1.63–1.84	1495	*32.1*	3170	*67.9*	1.63	1.52–1.73
**Lung**	**No**	121,547	*64.9*	65,825	*35.1*	1.00		64,412	*34.4*	122,960	*65.6*	1.00	
	**Yes**	6509	*35.1*	4947	*43.2*	1.38	1.32–1.43	3593	*31.4*	7863	*68.6*	1.18	1.31–1.23
**Heart**	**No**	124,548	*64.7*	67,963	*35.3*	1.00		65,601	*34.1*	126,910	*65.9*	1.00	
	**Yes**	3508	*55.5*	2809	*44.5*	1.35	1.28–1.42	2404	*38.1*	3913	*61.9*	1.26	1.19–1.33
**Hypertension**	**No**	111,723	*65.4*	59,135	*34.6*	1.00		57,273	*33.5*	113,585	*66.5*	1.00	
	**Yes**	16,333	*58.4*	11,637	*41.6*	1.21	1.17–1.24	10,732	*38.4*	17,238	*61.6*	1.17	1.14–1.21
**Kidney**	**No**	127,119	*64.5*	70,020	*35.5*	1.00		65,280	*34.8*	122,453	*65.2*	1.00	
	**Yes**	937	*55.5*	752	*44.5*	1.39	1.26–1.53	2725	*24.6*	8370	*75.4*	1.25	1.12–1.38
**Immune system**	**No**	118,074	*66.1*	63,240	*34.9*	1.00		65,145	*34.7*	122,775	*65.3*	1.00	
	**Yes**	9982	*57.0*	7532	*43.0*	1.21	1.17–1.25	2860	*26.2*	8048	*73.8*	1.23	1.19–1.27
**Tumors**	**No**	124,408	*64.6*	68,070	*35.4*	1.00		64,670	*34.9*	120,923	*65.1*	1.00	
	**Yes**	3648	*57.4*	2702	*42.6*	1.15	1.09–1.21	3335	*25.2*	9900	*74.8*	1.09	1.03–1.15
**Metabolic**	**No**	123,055	*64.7*	67,060	*35.3*	1.00		65,123	*35.0*	121,142	*65.0*	1.00	
	**Yes**	5001	*57.4*	3712	*42.6*	1.24	1.18–1.29	2882	*22.9*	9681	*77.1*	1.17	1.12–1.23
**Liver**	**No**	127,203	*64.5*	70,138	*35.5*	1.00		59,125	*35.7*	106,427	*64.3*	1.00	
	**Yes**	853	*57.4*	634	*42.6*	1.21	1.09–1.35	8880	*26.7*	24,396	*73.3*	1.18	1.06–1.31
**Depression and/or anxiety**	**No**	119,250	*65.0*	64,260	*35.0*	1.00		62,206	*34.6*	117,719	*65.4*	1.00	
	**Yes**	8806	*57.5*	6512	*42.5*	1.30	1.26–1.35	5799	*30.7*	13,104	*69.3*	1.34	1.29–1.39

§ Adjusted by sex, age, educational level, occupation.

## Data Availability

Data is available on request.

## Supplementary Materials

The following are available online at https://www.mdpi.com/1660-4601/18/6/3248/s1, Questionnaire 1

## Trial Registration

ClinicalTrials.gov NCT04471701; https://clinicaltrials.gov/ct2/show/NCT04471701, accessed on 12 February 2021.
