# Supplementary material for: Fear of COVID-19 for Individuals and Family Members: Indications from the National Cross-Sectional Study of the EPICOVID19 Web-Based Survey
Source: Int J Environ Res Public Health. 2021 Mar 21;18(6):3248. doi: 10.3390/ijerph18063248 (PMC8003842; doi:10.3390/ijerph18063248)
Supplement: Supplementary file 1 [file ijerph-18-03248-s001.pdf]

SOCIO-DEMOGRAPHIC DATA

**mandatory questions*

1. **Sex at birth*** M |__| F |__|

2. **Year of birth *** |__|__|__|__|

3. **Ethnicity***

|__| Europe
|__| Asia
|__| North America
|__| Central/South America
|__| Africa
|__| Oceania
|__| Other

4. **Postal code*** |__|__|__|__|__|

5. **Municipality*** |__|__|__|__|__|__|__|__|__|__|__|__|__|__|__|__|__|__|__|__|__|__|__|__|__|

6. **Education***

|__| None
|__| Primary school
|__| Middle high school
|__| High school
|__| University degree
|__| Post graduate degree (PhD, vocational master, medical specialization)

7. **Occupational status***

|__| Employed
|__| Student
|__| Unemployed
|__| Retired
|__| Other

8. **Last occupation***

|__| Armed forces occupations
|__| Managers
|__| Intellectual and scientific professionals (e.g. clinicians, engineers, researchers, teachers)
|__| Technicians and associate professionals (e.g. healthcare technicians, IT technicians)
|__| Clerk
|__| Service and sales workers (e.g. merchants, sales persons)
|__| Skilled agricultural and fishery workers
|__| Craft and related trades workers
|__| Plant and machine operators, assemblers, and drivers of vehicles
|__| Other

CLINICAL EVALUATION

9. Did you have one or more of the following symptoms since the 1st of February 2020? *

|__| Fever with a temperature greater than 37.5 °C for at least three consecutive days
|__| Cough
|__| Sore throat/rhinorrea
|__| Headache
|__| Myalgia
|__| Olfactory or taste disorders
|__| Shortness of breath
|__| Chest pain
|__| Feelings of having a fast-beating
|__| Gastrointestinal disorders (diarrhoea, nausea, vomiting)
|__| Conjunctivitis
|__| Pneumoniae

10. If you had at least one of the symptoms above, please indicate in which month they occurred for the first time *

|__| February
|__| March
|__| April

11. Have you ever been diagnosed with one or more of the following conditions?

|__| Lung diseases (e.g. asthma, obstructive pulmonary disease)
|__| Heart diseases (e.g. ischemic heart disease, atrial fibrillation)
|__| Hypertension
|__| Kidney diseases
|__| Immune system diseases (e.g. thyroid disease, psoriasis, rheumatoid arthritis)
|__| Tumours
|__| Metabolic diseases (e.g. diabetes, obesity, gout)
|__| Liver diseases (e.g. hepatitis, cirrhosis, liver failure)
|__| Depression and/or anxiety

12. Please indicate other conditions

|__| Surgical procedures under general anaesthesia during the last year
|__| Transplants
|__| Allergies
|__| Pregnancy
|__| Non self-sufficient in carrying out daily activities
|__| Healthcare workers (e.g. clinicians, nurses, rescuer, pharmacist)

13. Did you carry out the following vaccinations?*

- Flu shot during the last autumn |__| Yes |__| No
- Anti-pneumococcal in the last 12 months |__| Yes |__| No
- Other vaccinations in the last 12 months |__| Yes |__| No

14. Do you regularly take one or more of the following medicines?

|__| Aspirin
|__| Anti-hypertensive
|__| Hypocholesterolemic drugs
|__| Anti-diabetics
|__| Anti-cancer drugs
|__| Corticosteroids
|__| Thyroid drugs (e.g. euthyrox)
|__| Anti-inflammatory drugs
|__| Anxiety medications and/or sedatives
|__| Anti-depressant
|__| Supplements (e.g. vitamins)

If females

15. Have you ever been taking birth pills and/or hormone replacement therapy?*

|__| No
|__| Yes, in the past, for less than 5 years
|__| Yes, in the past, for more than 5 years
|__| Yes currently, taking it less than 5 years
|__| Yes currently, taking it more than 5 years

16. Indicate the number of completed pregnancies*

|__| 0
|__| 1
|__| 2
|__| 3 or more

17. Have you been in a close contact (direct contact at a distance of less than 2 meters, or in a closed environment such as a house, workplace, transportation vehicles) with confirmed COVID-19 cases, live or deceased?*

|__| Yes |__| No

18. Have you been in a close contact (direct contact at a distance of less than 2 meters, or in a closed environment such as a house, workplace, transportation vehicles) with suspected COVID-19 cases, live or deceased?*

|__| Yes |__| No |__| I don't know

19. Did you contact the emergency number and/or the general practitioner to report any symptoms of suspected infection by COVID-19?*

|__| No
|__| No but I went to the hospital on my own initiative
|__| Yes, and they suggested to me isolation
|__| Yes, and they did not suggest to me isolation
|__| Yes, and I was sent to the hospital

20. Have you been tested for COVID-19? *

|__| Yes, with a positive result
|__| Yes, with a negative result
|__| Yes, but I do not know the result
|__| No, I did not perform any test

21. Have you been hospitalized due to COVID-19, either as a suspected or as a confirmed case?*

|__| Yes |__| No

22. Please indicate other elements that might be of relevance for COVID-19

____

____

PERSONAL CHARACTERISTICS AND HEALTH STATUS

23. How would you describe your health in general?*
|__| Very bad |__| Bad |__| Adequate |__| Good |__| Very good

24. Do you fear getting infected with the coronavirus (COVID-19)?*

|__| No
|__| Just a little bit
|__| Neutral
|__| Quite enough
|__| Yes, a lot

25. Do you fear your family being infected with the coronavirus (COVID-19)?*

|__| No
|__| Just a little bit
|__| Neutral
|__| Quite enough
|__| Yes, a lot

HOUSING CONDITIONS

26. Your home is located in:*
|__| City centre with more than 100.000 inhabitants
|__| Suburbs of cities with more than 100.000 inhabitants
|__| Small town
|__| Countryside

27. Your home is located in an area where the road traffic is:*
|__| Intense (living near a busy road)
|__| Moderate
|__| Low

28. How many rooms there are in your home (excluding the bathroom and auxiliary spaces)?*
|__| One |__| Two |__| Three |__| More than three

29. Besides you, how many people live in your household?*
|__| None |__| One |__| Two |__| More than two

30. Are there in the same household elderly persons or anyone with immunocompromising or chronic disease conditions?*
|__| Yes |__| No

LIFESTYLE

31. How many people have you been in contact with in average, prior to the Governmental restrictions on lockdown?*
|__| Less than 10 |__| Between 10 and 100 |__| More than 100

32. Do you smoke?*
|__| I have never smoked or I smoked less than 100 cigarettes in my lifetime

|__| I am a former smoker (I have smoked at least 100 cigarettes in my lifetime and I do not smoke anymore)
|__| Yes, smoking less than 10 cigarettes per day
|__| Yes, smoking between 10 and 20 cigarettes per day
|__| Yes, smoking more than 20 cigarettes per day (more than one pack per day)

If former smoker
33. How many year have you smoked?* |__|__|

If current smoker
34. How many years?* |__|__|

35. Prior to the lockdown by the Governmental restrictions, for how long did you follow a regular routine of moderate or intense physical activity (swimming, racing)?*
|__| Not doing any physical activity or doing it for less than 10 minutes per week
|__| Between 10 minutes and two hours and half per week
|__| More than two hours and half per week

BEHAVIOURS FOLLOWING THE LOCKDOWN

36. Please indicate your employment status following the restrictions introduced by the Italian Government since March, 9th 2020 *
|__| Continued going to work at my workplace
|__| Working from home
|__| I had to stop working due to the emergency
|__| I am not employed

37. Since March, 9th 2020, how many times do you go out during a week? *
|__| Never
|__| 1
|__| 2-3
|__| 4-5
|__| 6 or more

38. Since March, 9th 2020, do you use public transport go to work or to provide supplies*
|__| No, never
|__| Yes, 1-3 times per week
|__| Yes, 4-6 times per week
|__| Yes, 7 or more times per week

Any additional information and comments

____
____
____
